# *C1R* Mutations Trigger Constitutive Complement 1 Activation in Periodontal Ehlers-Danlos Syndrome

**DOI:** 10.3389/fimmu.2019.02537

**Published:** 2019-11-05

**Authors:** Rebekka Gröbner, Ines Kapferer-Seebacher, Albert Amberger, Rita Redolfi, Fabien Dalonneau, Erik Björck, Di Milnes, Isabelle Bally, Veronique Rossi, Nicole Thielens, Heribert Stoiber, Christine Gaboriaud, Johannes Zschocke

**Affiliations:** ^1^Institute for Human Genetics, Medical University Innsbruck, Innsbruck, Austria; ^2^Department for Operative and Restorative Dentistry, Medical University Innsbruck, Innsbruck, Austria; ^3^University of Grenoble Alpes, CEA, CNRS, IBS, Grenoble, France; ^4^Department of Molecular Medicine and Surgery, Karolinska Institute, Stockholm, Sweden; ^5^Department of Clinical Genetics, Karolinska University Hospital, Stockholm, Sweden; ^6^Genetic Health Queensland, Royal Brisbane and Women's Hospital, Herston, QLD, Australia; ^7^Institute of Virology, Medical University Innsbruck, Innsbruck, Austria

**Keywords:** complement system, connective tissue, periodontitis, C1r/s, Ehlers-Danlos syndrome

## Abstract

Heterozygous missense or in-frame insertion/deletion mutations in complement 1 subunits C1r and C1s cause periodontal Ehlers-Danlos Syndrome (pEDS), a specific EDS subtype characterized by early severe periodontal destruction and connective tissue abnormalities like easy bruising, pretibial haemosiderotic plaques, and joint hypermobility. We report extensive functional studies of 16 *C1R* variants associated with pEDS by *in-vitro* overexpression studies in HEK293T cells followed by western blot, size exclusion chromatography and surface plasmon resonance analyses. Patient-derived skin fibroblasts were analyzed by western blot and Enzyme-linked Immunosorbent Assay (ELISA). Overexpression of *C1R* variants in HEK293T cells revealed that none of the pEDS variants was integrated into the C1 complex but cause extracellular presence of catalytic C1r/C1s activities. Variants showed domain-specific abnormalities of intracellular processing and secretion with preservation of serine protease function in the supernatant. In contrast to C1r wild type, and with the exception of a *C1R* missense variant disabling a C1q binding site, pEDS variants had different impact on the cell: retention of C1r fragments inside the cell, secretion of aggregates, or a new C1r cleavage site. Overexpression of *C1R* variants in HEK293T as well as western blot analyses of patient fibroblasts showed decreased levels of secreted C1r. Importantly, all available patient fibroblasts exhibited activated C1s and activation of externally added C4 in the supernatant while control cell lines secreted proenzyme C1s and showed no increase in C4 activation. The central elements in the pathogenesis of pEDS seem to be the intracellular activation of C1r and/or C1s, and extracellular presence of activated C1s that independently of microbial triggers can activate the classical complement cascade.

## Introduction

Periodontitis is a common chronic disease caused by a dysbalance between the oral microbiome and the host immune response leading to inflammatory destruction of the tooth-supporting tissues and finally loss of teeth. Periodontal Ehlers-Danlos Syndrome (pEDS) (OMIM 130080 and 617174, prevalence: unknown) is an autosomal dominant type of Ehlers-Danlos Syndrome (EDS) (1, 2) characterized by early severe and rapidly progressing periodontal destruction and variable connective tissue abnormalities (3). Clinical features include lack of attached gingiva already present in childhood, easy bruising, pretibial haemosiderotic plaques, doughy skin, hoarse voice, poor wound healing, and hypermobility of joints. We recently showed that pEDS is caused by heterozygous missense or in-frame insertion/deletion mutations in *C1R* or *C1S* (4). These genes code for complement 1 subunits C1r and C1s, serine proteases that play a key role in the innate immune response. The penetrance in the individuals with pEDS identified up to now is 100%, and there is no clinical evidence for relevant modifier genes.

**Figure 1:**
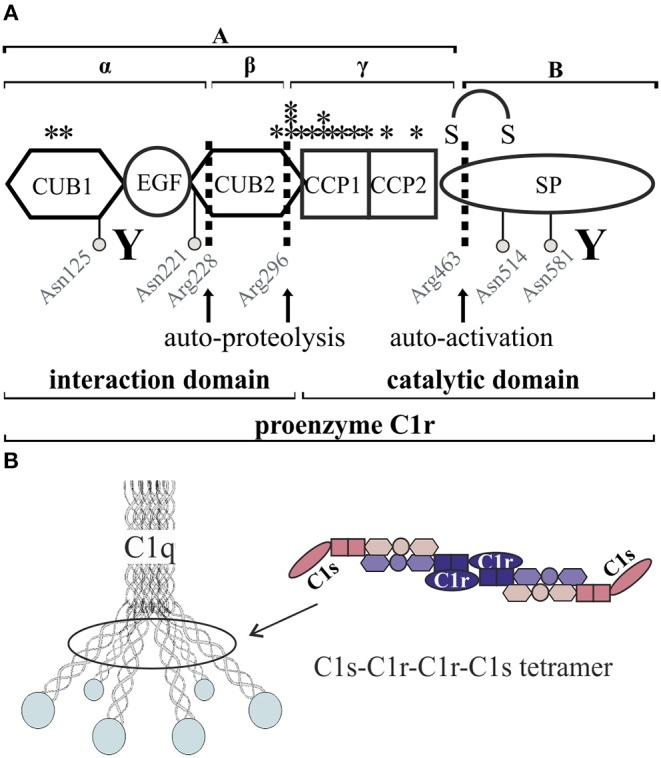
Schematic overview of C1r domain structure and secretion pattern. **(A)** Cleavage sites (arrows) as well as glycosylation sites (gray circles) are marked. Investigated pEDS variants (see also Table 2) are marked with stars. CUB1-EGF-CUB2 is described as the interaction domain and CCP1-CCP2-SP as the catalytic domain of C1r. “Y” indicates N- and C-terminal antibody target regions used in this study. Full-length proenzyme C1r has a molecular mass of ~100 kDa on western blot. Activation occurs through cleavage at Arg463, which produces the disulfide-linked A- and B-chains with apparent molecular masses when analyzed by SDS-PAGE under reducing conditions of 55 and 38 kDa. C1r is also known to undergo two additional auto-proteolytic cleavages at Arg 228 and 296 in the A-chain that produce an N-terminal α-fragment with an apparent mass of 35 kDa, a β-fragment, and a γ-fragment disulfide-linked to the B-chain (5, 6). The fragments β and γ cannot be detected under reducing conditions by the C1r antibodies used in this study. **(B)** The C1 complex consists of a C1r_2_-C1s_2_ tetramer embedded into the umbrella-like hexamer of C1q heterotrimers.

C1r and C1s have a similar protein domain structure with the N-terminal interaction domain including CUB (complement C1r/C1s, Uegf, Bmp1) and EGF (epidermal growth factor) modules in a CUB1-EGF-CUB2 arrangement, two complement-control-protein modules CCP1 and CCP2, and a serine protease (SP) domain (Figure 1A, Table 1). Two molecules of C1r and C1s form a tetramer which binds to the collagen-like stalks of C1q assembled from six heterotrimers to build up the C1 complex (Figure 1B). Binding of the resulting C1 complex to activating targets such as antibody-antigen complexes causes C1q conformation changes which trigger auto-activation of C1r. This involves cleavage of the protein between the N-terminal A-chain and the C-terminal B-chain; both chains remain linked through a disulfide bridge. C1r activation causes cleavage of C1s at a similar position, and subsequently activation of C4 and C2, ultimately resulting in activation of the central complement protein C3 (7, 8). Activation of C3 and downstream signaling pathways triggered by host-microbe interactions has been shown to promote inflammatory bone loss in periodontitis (9). Based on the results from a C3-knock-out mouse model (10), C3-targeted drug candidates have been suggested as novel immunotherapeutics for periodontal disease (11). Over-activation of the complement system causes periodontitis as proposed by several pre-clinical studies and clinical case reports and was reviewed elsewhere (12).

**Table 1:** Summary of all C1r fragments with their predicted and apparent mass.

**Amino** **acid**	**Description**	**Predicted polypeptide** **chain mass (kDa)**	**Number of N-linked sugars**	**Apparent mass on** **western blot (kDa)**
18–463	A (heavy) chain	51	2	55
18–228	α-fragment	28	2	35
229–296	β-fragment	7.7	–	Not detected
18–296	αβ-fragment	35.7	2	40
297–449	γ-fragment	19.6	–	Not detected
464–705	B (light) chain	27	2	38
18…705	Proenzyme C1r	78	4	100

Pathogenic *C1R* and *C1S* variants in pEDS are expected to be gain-of-function variants as heterozygous loss-of-function variants in these genes are asymptomatic while homozygous variants cause systemic lupus erythematosus without periodontitis (13–15). However, the exact gain of function mechanism has not yet been characterized. Homozygosity for pEDS causing variants has not been observed. All *C1R* and *C1S* pEDS variants identified so far are located in the A-chain, mostly CCP1 and CCP2. In order to elucidate the pathomechanism underlying pEDS we systematically investigated the molecular consequences of all 16 *C1R* variants known at the onset of the study.

## Materials and Methods

### Patient-Derived Skin Fibroblasts and C4a ELISA

Skin fibroblast samples were obtained after informed written consent from pEDS patients in four families with *C1R* variants c.149_150TC>AT (p.V50D; *n* = 3), c.277G>T (p.G93C; *n* = 1), c.902G>C (p.R301P; *n* = 2), and c.926G>T (p.C309F; *n* = 1). All pathogenic variants identified were specific for individual families, without evidence of recurrence for any pEDS variant. We did not identify any family with a *de-novo* mutation although parental samples were not available for all affected individuals. Fibroblasts from healthy individuals served as controls.

Fibroblasts were cultured at 37°C/5% CO_2_ in DMEM (Sigma Aldrich Chemie GmbH, Schnelldorf, Germany, #D5546-500ML) supplemented with 10% fetal bovine serum (FBS) (Gibco, Fisher Scientific GmbH, Vienna, Austria, #10500-064), penicillin/streptromycin (Sigma Aldrich Chemie GmbH, #P4333), and 2 mM L-glutamine (Lonza, Szabo-Scandic, Vienna, Austria, #BE17-605E). RNA was extracted with RNeasy Kit (Qiagen, Hilden, Germany, #74104) according to manufacturer's instructions for validation of heterozygous expression of mutated *C1R*. RNA was reverse transcribed (Fermentas, Distributor: Fisher Scientific Austria GmbH, #K1652) followed by PCR-based amplification of *C1R* and Sanger sequencing.

Medium was exchanged from DMEM to Ex-Cell 293 serum-free cell culture medium for HEK293T cells (Sigma Aldrich Chemie GmbH, #14571C-1000ML) supplemented with 2 mM L-glutamine 48–72 h prior to harvest of supernatant. Exchange to serum-free medium was necessary to prevent detection of serum-derived complement proteins. Cells were harvested separately and lysed for 10 min on ice in RIPA buffer (50 mM Tris-HCl pH 7.4, 0.154 M NaCl, 1 mM EDTA, 0.5% Triton X-100, 0.05% SDS) containing protease inhibitor (Fisher Scientific Austria GmbH, #87786). Protein concentration of cell lysates was determined by Bradford assay (Biorad Laboratories GmbH, Vienna, Austria, #500-0006). Harvested cell culture supernatants were concentrated 10-fold with Pierce Protein Concentrator (Fisher Scientific Austria GmbH, #88517). Protein concentration of cell culture supernatants was normalized based on the protein concentration of the respective cell lysates. Cells and supernatants were stored at −20°C until further analyses.

Enzyme-linked Immunosorbent Assay (ELISA) based quantification of C4a (Quidel, Distributor: Biomedica Medizinprodukte GmbH & Co KG, Vienna, Austria, #A036) concentrations was conducted with concentrated cell culture supernatants following the supplier's instructions. C4 purified from human serum (Sigma Aldrich, #C8195) was added at a final concentration of 2.2 ng/ml to harvested cell culture supernatant. Fibroblasts express C1r, C1s and C1 inhibitor (C1Inh) but not C1q or C4; qPCR analysis of fibroblasts showed no difference in the amounts of transcripts of *C1R, C1S*, and *SERPING1* (coding for C1Inh) between patients and controls (data not shown). Standard curve as well as samples were measured as duplicates. Statistical analyses were performed with GraphPad Prism 7.00. Results were tested for normality with D-Agostino-Pearson omnibus normality test. Since not all sample groups passed the normality test, we applied the unpaired, non-parametric Mann-Whitney test (significance level α = 0.05; ^*^ ≤ 0.05; ^**^ ≤ 0.01; ^***^ ≤ 0.001). C4a levels were determined in supernatant of cultured fibroblasts and serum from individuals with *C1R* c.149_150TC>AT (p.V50D), *C1R* c.902G>C (p.R301P), and *C1R* c.926G>T (p.C309F). Cell culture supernatant was harvested and stored as described above. Serum samples were immediately centrifuged and frozen to −80°C; there were no differences in sample handling between patients and controls.

### HEK293T Cells as *in-vitro* Overexpression Model of C1r Variants

A total of 16 different *C1R* variants from two publications (4, 16) were investigated, including one previously unpublished variant c.277G>T (p.G93C) identified in a family from Australia (Table 2). All variants are predicted to be pathogenic, and none of the variants has been found in registered control populations [ExAc database http://exac.broadinstitute.org/ (17), last accessed September 26, 2019]. Variant p.G93C is the only novel variant in the present study; it is not recorded in variant databases (e.g., GnomAD) and is predicted to be probably damaging by PolyPhen. In addition, we studied two missense variants recorded in control samples in the ExAc database. The mutation type and location could be compatible with a functional effect but both variants were predicted to be benign by variant assessment programs such as PolyPhen, and no association with pEDS was reported. One of the variants (c.547C>T, p.(E183K)) has a very high minor allele frequency of 0.1174, precluding a strong unknown functional effect, the other variant (c.902G>A, p.(R301C)) is very rare (minor allele frequency 0.00006612) but is at the same position as a disease-causing variant identified by us in a pEDS patient. We expected that overexpression of these variants should be indistinguishable from wild type.

**Table 2:** List of all *C1R* variants analyzed in this study.

**DNA (c.) (GRCh38)**	**Protein (*p*.)**	**Domain**	**MAF**	PolyPhen/^*^PROVEAN prediction	**ClinVar**
c.149_150TC>AT	p.Val50Asp	CUB1	Not listed	prob.dam (1.000)	VCV000372130
c.277G>T	p.Gly93Cys	CUB1	Not listed	prob.dam (0.986)	VCV000597277
c.869A>G	p.(Asp290Gly)	C1q-binding	Not listed	prob.dam (0.987)	VCV000267356
c.890G>A	p.(Gly297Asp)	CUB2	Not listed	prob.dam (1.000)	VCV000372129
c.899T>C	p.(Leu300Pro)	CUB2	Not listed	prob.dam (0.997)	VCV000267354
c.902G>C	p.Arg301Pro	CUB2	Not listed	poss.dam (0.719)	VCV000267352
c.905A>G	p.(Tyr302Cys)	CUB2	Not listed	prob.dam (1.000)	VCV000375577
c.917_927delinsGGACA	p.(Ile306_Cys309delinsArgArg)	CCP1	Not listed	deleterious^*^ (−15.383)	VCV000267355
c.927C>G	p.(Cys309Trp)	CCP1	Not listed	prob.dam (1.000)	VCV000267353
c.926G>T	p.Cys309Phe	CCP1	Not listed	prob.dam (0.996)	Not available
c.1012T>C	p.(Cys338Arg)	CCP1	Not listed	prob.dam (1.000)	VCV000375578
c.1073G>T	p.(Cys358Phe)	CCP1	Not listed	prob.dam (1.000)	VCV000267351
c.1092G>C	p.(Trp364Cys)	CCP1	Not listed	prob.dam (1.000)	VCV000375579
c.1113C>G	p.(Cys371Trp)	CCP1	Not listed	prob.dam (1.000)	VCV000375580
c.1200_1215delinsTCATGTAATA	p.(Arg401_Tyr405delinsHisValIle)	CCP2	Not listed	deleterious^*^ (−19.321)	VCV000375581
c.1303T>C	p.Trp435Arg	CCP2	Not listed	prob.dam (1.000)	VCV000375582
c.547C>T	p.(Glu183Lys)	EGF-like	0.1174	benign (0.006)	Not available
c.902G>A	p.(Arg301Cys)	CUB2	0.00006612	benign (0.397)	Not available

*All variants have been identified as disease-causing in pEDS patients except that last two variants c.547C>T and c.902G>A which we selected as control variants. MAF, minor allele frequency (ExAc database)*.

“*” *indicates prediction with PROVEAN Protein tool (http://provean.jcvi.org/index.php). prob.dam, probably damaging; poss.dam, possibly damaging*.

Finally, we generated enzymatically inactive C1r WT and variants by adding a previously designed active site mutation p.S654A to WT and variant clones (18). This variant removes the active center Serine residue which is essential for the serine protease function. Under physiological conditions in plasma, proenzyme activation of normal C1r is prevented by presence of C1Inh. Variant p.S654A precludes auto-activation, also in the absence of C1Inh. It is not known whether p.S654A interferes with C1Inh; however, this is irrelevant with regard to the loss of function effect of the variant.

*C1R* WT was cloned into the transfection vector pcDNA3.1(+)NEO (GenScript, Distributor: Hölzel Diagnostika Handels GmbH, Köln, Germany), and all variants were inserted by site-directed mutagenesis (Peqlab, Distributor: VWR, Vienna, Austria, #07-KK2100-01) according to manufacturer's instructions. C1s WT was cloned into a p3xFLAG-CMV14 vector. HEK293T cells (Sigma Aldrich Chemie GmbH, Schnelldorf, Germany) were cultured at 37°C/5% CO_2_ in EMEM (Sigma Aldrich Chemie GmbH, #M2279-500 ML) supplemented with 10% FBS, penicillin/streptomycin and 2 mM L-glutamine. For transfection purposes, 1 × 10^6^ cells were seeded into T-25 flasks the day before transfection. Immediately before transfection, cells were washed with PBS and medium was exchanged to EMEM without FBS. Transfection was conducted with 12 μl Turbofect (Fisher Scientific GmbH, #R0531) and 4 μg of *C1R* plasmid in 1 ml EMEM. Five hours after transfection cell medium was exchanged to EMEM supplemented with FBS and penicillin/streptomycin. The day after transfection, medium was exchanged to Ex-Cell 293 serum-free culture medium supplemented with 2 mM L-glutamine. Cells and supernatants were harvested 48 h after transfection, as described for patient-derived skin fibroblasts.

In order to determine the ability of C1r to cleave C1s, concentrated cell culture supernatants of single transfected HEK293T cells containing C1r (WT or variant) were mixed 1:1 with supernatant of C1s transfected cells. Supernatant mixtures were incubated for 1 h at 37°C and analyzed by western blot (C1s antibody).

### Western Blot Analyses

Western blot analyses were performed under denaturing and reducing conditions (unless indicated otherwise) using standard procedures. Protein samples were separated by using a 10% SDS gel (Biorad Laboratories GmbH, #4561031) and running buffer (20 mM Tris, 0.2 M glycine, 3.5 mM SDS). Twenty microgram of whole cell lysate were used and supernatant normalized to according cell pellet. Page Ruler prestained protein ladder (Fisher Scientific GmbH, #26616) was used for determining protein molecular weight. Proteins were transferred onto a PVDF membrane via wet blotting (1 h, 100 V, 4°C). Membranes were probed with C1r or C1s specific antibodies (Abcam plc, Cambridge, UK; C1r: N-terminal #ab71652 or C-terminal #ab185212; C1s: #ab155270) diluted 1:2,000 in PBS-0.05% Tween 20 (PBS-T) containing 5% milk (w/v). HRP-conjugated secondary antibody (Dako, Glostrup, Denmark, #P0448) was used 1:10,000 in 5% milk/PBS-T. Protein bands were detected with ECL (GE Healthcare, Vienna, Austria, #RPN3243) and developed on X-ray films (Amersham Hyperfilm ECL, Distributor: VWR, Vienna, Austria). For overexpressed C1r variants, empty vector transfected HEK293T cells were used as negative control.

### Size Exclusion Chromatography of the C1s-C1r-C1r-C1s Tetramer

Proenzyme C1s-C1r-C1r-C1s tetramers containing C1r WT or variant with additional p.S654A mutation were produced by co-transfected 293-F cells and purified as described (19). Size exclusion chromatography of the purified tetramers was performed by using a Superose 6 Increase 10/300 GL column (GE Healthcare, UGAP, Marne la Vallée, France) equilibrated in 50 mM Tris, 150 mM NaCl, pH 7.4 containing 2 mM CaCl_2_ or 5 mM EDTA at a flow rate of 0.5 ml/min.

### Surface Plasmon Resonance (SPR) Analyses

Binding of the C1r-C1s tetramer to immobilized C1q was analyzed by SPR using a Biacore T200 instrument (GE Healthcare). Plasma derived C1q [purified according to Arlaud et al. (20)] was diluted at 35 μg/ml in 10 mM sodium acetate pH 5 and immobilized on a Series S CM5 sensor chip (GE Healthcare) using the amine coupling chemistry in 10 mM Hepes, 150 mM NaCl, EDTA 3 mM pH 7.4, surfactant P20 0.05% (HBS-EP+, GE Healthcare). A flow cell submitted to the coupling steps without immobilized protein was used as blank. Binding of the tetramer was measured by injecting 90 μl of each tetramer (5 nM) at a flow rate of 30 μl/min in 50 mM Tris-HCl, 150 mM NaCl, 2 mM CaCl_2_, 0.05% Tween 20, pH 7.4 followed by 300 s dissociation. The specific binding signal was obtained by subtracting the signal over the blank surface. Regeneration was achieved by a 15 μl injection of 1 M NaCl, 10 mM EDTA, pH 7.4.

### Production and Protein Fragment Analysis of the p.W435R Variant of C1r

Recombinant p.W435R variant of C1r was produced in the FreeStyle 293 Expression System (Thermo Fisher), using a pcDNA3.1/Neo(+) plasmid encoding human C1r with a C-terminal Strep-tag. 293-F cells grown in FreeStyle 293 medium were transfected with this plasmid using 293 fectin and stable transfectants were selected with 400 μg/ml neomycin (Thermo Fisher). Recombinant C1r was purified from the culture supernatant by chromatography on StrepTactin Sepharose High performance (Sigma-GE28-9355-99), as recommended by the manufacturer. Briefly, the cell culture supernatant was concentrated 6-fold, dialyzed against 100 mM Tris, 150 mM NaCl, 1 mM EDTA, pH 8.0, and applied to a 1-ml column equilibrated in the same buffer. Elution was carried out using 2.5 mM desthiobiotin (Sigma-Aldrich) in the same buffer. The eluted protein was dialyzed in the same buffer and concentrated to 0.3–0.45 mg/ml. N-terminal sequence determination of the major band observed after SDS-PAGE analysis under non-reducing conditions was performed using an Applied Biosystems gas-phase sequencer model 492 coupled online with an Applied Biosystems Model 140C HPLC system.

## Results

### pEDS Variants Cause Domain-Specific Abnormalities of C1r Processing and Secretion

**Figure 2:**
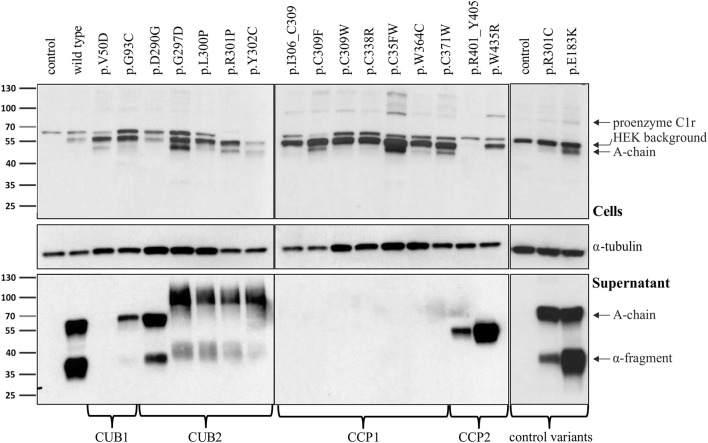
Domain-specific secretion patterns of C1r variant proteins. HEK293T cells were transiently transfected with C1r WT or variants (“control” = empty vector). Cell lysates (upper panel) and supernatants (lower panel) were used for western blot with an N-terminal anti-C1r antibody 48 h after transfection under reducing conditions. This antibody may specifically visualize (1) the full-length C1r (100 kDa); (2) the A-chain (55 kDa) after auto-activation of C1r; or (3) the α-fragment (35 kDa) generated via an auto-proteolytic event after secretion of the activated protein. An intracellular HEK-specific background band is seen at around 60 kDa. Little full-length C1r (100 kDa) and presence of A-chain was detected in all cell lysates. Most variants resulted in increased intracellular presence of A-chain fragments, sometimes with an additional smaller C1r fragment that may represent a degradation product. Overexpression of C1r WT, the control variants p.(R301C) and p.(E183K), and the variant p.(D290G), resulted in high amounts of A-chain and α-fragment in the supernatant. All other variants show domain-specific abnormalities of intracellular processing and secretion. Strongly reduced extracellular secretion was found for variants in the CUB1 and CCP1 domains. All CUB2 variants (except p.(D290G)) show abnormal protein aggregates in the supernatant, the exact nature of which cannot be specified. Abnormal protein fragments without proteolysis were found for CCP2 variants.

**Figure 3:**
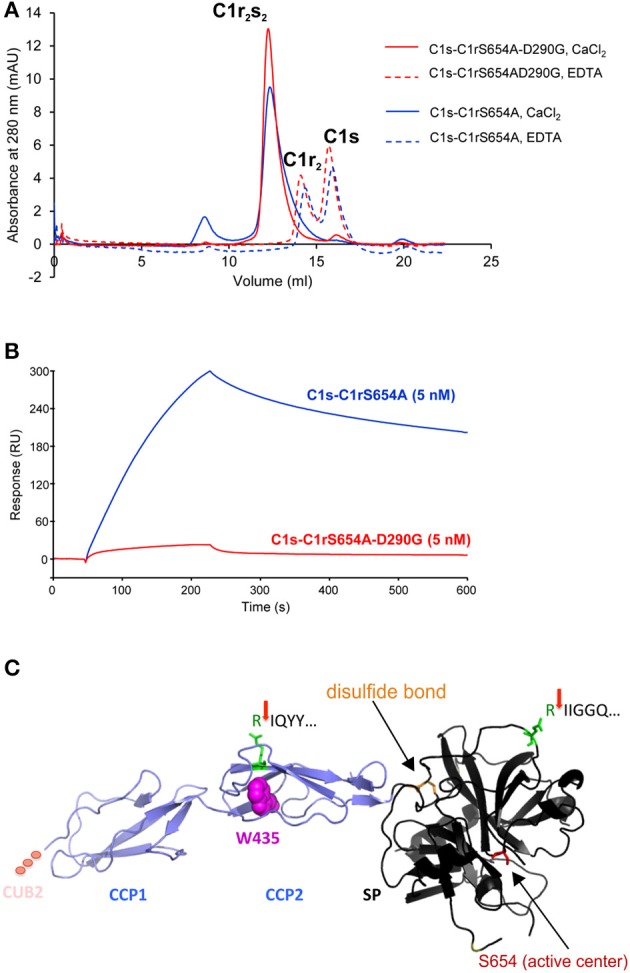
C1r p.(D290G) variant strongly inhibits binding of the tetramer to C1q whereas C1r p.W435R induces a new cleavage site. **(A)** Size exclusion chromatography of the tetramers (containing C1r p.S654A with/without p.(D290G)) in the presence of CaCl_2_ (plain lines) or EDTA (dotted lines). WT and variant C1r yielded similar major peaks in the presence of CaCl_2_. In the presence of EDTA, the tetramer was dissociated into two later eluting peaks, corresponding to the C1r dimer and C1s monomer. **(B)** SPR analysis of the binding of the tetramers to immobilized C1q (14,300 RU). Ninety microliter of each tetramer (5 nM) were injected at a flow rate of 30 μl/min followed by 300 s dissociation. C1r p.(D290G) allows formation of the C1s-C1r-C1r-C1s tetramer but strongly inhibits binding of the tetramer to C1q. **(C)** N-terminal sequencing of C1r p.W435R identified two cleavage sites: IQYY (new cleavage) and IIGGQ (activation cleavage) indicated by red arrows. The preceding Arg is highlighted in green. The 3D structure shows proximity between p.W435R and the new cleavage site. The two cleavage sites correspond to C1r specificity (cleavage between Arg and Ile). In the SP domain disulfide bond maintaining integrity after activation cleavage (orange) and active serine (red) are marked (PDB ID 1GPZ).

Intracellular processing and secretion of C1r WT and 16 different C1r variants (Table 2) was studied by transient overexpression in HEK293T cells (which do not express C1r/s or C1q) using western blot analysis of cell lysates and supernatants. The results obtained by an antibody against the N-terminal domain of C1r are depicted in Figure 2. Because of auto-activation in the absence of C1 inhibitor (C1Inh; Gene: SERPING1), little full-length C1r (100 kDa) but presence of A-chain was detected in all cell lysates. Variants resulted in intracellular presence of A-chain, sometimes with an additional smaller C1r fragment that may represent a degradation product or reduced glycosylation. Overexpressed C1r WT is effectively secreted into the supernatant where it is visible either as the auto-activated A-chain (55 kDa), or the α-fragment (35 kDa) generated via an auto-proteolytic event (5, 6). The same WT activation and secretion pattern was observed for the two control variants p.(R301C) and p.(E183K), as expected. In contrast, all pEDS-related C1r variants except C1r p.(D290G) showed evidence of intracellular accumulation as shown by an increased intracellular signal of activated C1r A-chain, and abnormal secretion patterns as follows (Figure 2):

1. The N-terminal anti-C1r antibody showed strongly reduced signals for CUB1 variants p.V50D (no bands) and p.G93C (reduced A-chain, absent α-fragment) in the supernatant under standard conditions. Increased loading volume and exposure time during detection revealed traces of A-chain and α-fragment also for p.V50D.
2. Variants in the CUB2 domain (except p.(D290G)) resulted in two fuzzy bands of molecular weight higher than WT (42 kDa and 100 kDa, respectively) that may represent aggregates formed by C1r A-chains. These fragments were not visualized by a C-terminal anti-C1r antibody, confirming that they did not represent full-length C1r.
3. Secretion of C1r with the CUB2 p.(D290G) variant was similar to C1r WT. Size exclusion chromatography showed that C1r WT and p.(D290G) yielded similar major peaks in the presence of CaCl2 indicating that variant proteins form C1s-C1r-C1r-C1s tetramer like C1r WT (Figure 3A). In the presence of EDTA, the tetramer was dissociated into two later eluting peaks, corresponding to the C1r dimer and C1s monomer. However, SPR analyses revealed that the C1r p.(D290G) variant strongly inhibits binding of the tetramer to C1q and forming of a functional C1 complex (Figure 3B). Similar results were observed previously for the C1r p.D290A variant (21).
4. CCP1 variants were absent in the supernatant under normal conditions. Increased exposure time revealed traces of abnormal fragments (comparable to pattern 2) that did not represent full-length when detected by a C-terminal antibody.
5. CCP2 variants yielded an N-terminal fragment (45 kDa) slightly shorter than the A-chain that does not give rise to an α-fragment and does not seem to undergo auto-proteolytic cleavage. In order to determine the origin of the shorter fragments found for these variants, the recombinant p.W435R variant was overexpressed, partially purified and N-terminally sequenced. This identified a new cleavage site at Arg401 of CCP2 leading to an abnormal A-chain shortened by 62 amino acids. The 3D structure shows proximity between p.W435 and the new cleavage site (Figure 3C). The two cleavage sites correspond to C1r specificity (cleavage between Arg and Ile).

### Pathogenic C1r Variants Are Associated With Secretion of Active Serine Protease

**Figure 4:**
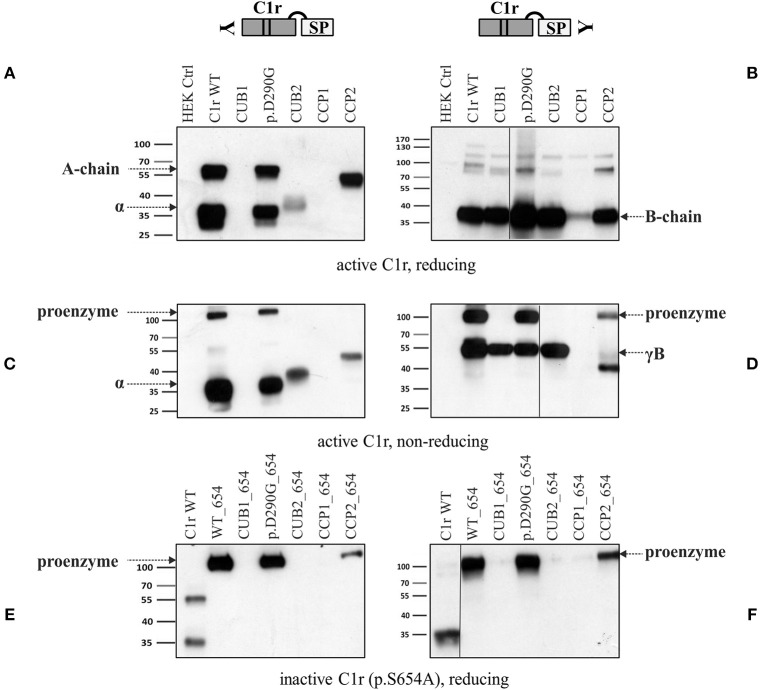
Secretion of C1r active serine protease for all C1r variants. HEK293T cells were transiently transfected with C1r WT or variant as enzymatically active or inactive (additional mutation p.S654A) form (“HEK Ctrl” = empty vector). Supernatant 48 h post-transfection was used for western blot with N- and C-terminal antibodies under reducing or non-reducing conditions as indicated. One mutation of each domain was chosen: CUB1 (p.V50D), C1q-binding site (p.(D290G)), CUB2 (p.(L300P)), CCP1 (p.(C338R)), CCP2 (p.W435R). **(A)** Domain-specific secretion pattern of N-terminal fragments. **(B)** C1r WT and all variants show secretion of catalytic B-chain. **(C)** Shows same domain-specific pattern as in **(A)** with expected differences (disulfide-linked chains running as full-length protein) under non-reducing conditions. **(D)** Presence of full-length in WT, p.(D290G) and CCP2. The γB-fragment is present in WT and all variants with the exception of CCP1. The new cleavage site in CCP2 variant results in smaller fragment size. **(E,F)** Enzymatically inactive C1r variants were secreted for WT, p.(D290G), and CCP2. Other variants were not detected in the supernatant.

Supernatants of representative variants from each domain were investigated in more detail under reducing and non-reducing conditions using specific N- and C-terminal anti-C1r antibodies (Figure 4 and Figure S1). These analyses confirmed the previously observed secretion patterns under reducing conditions (Figure 4A). The B-chain, detected by the C-terminal antibody was present in the supernatants of C1r WT and all C1r variants studied (Figure 4B). The investigated CCP1 variant (p.(C338R)) gave a very weak signal in western blots in repeated analyses, indicating reduced or slow secretion of this variant. Under non-reducing western blot conditions (Figures 4C,D), full-length protein was strongly present in the supernatant for WT and variant p.(D290G). Smaller amounts of full-length protein were found for the CCP2 variant (p.W435R) that gives rise to an abnormal cleavage site (this was only shown by the C-terminal antibody which is more sensitive for full-length C1r than the N-terminal antibody, see also Figures 4C,D). The serine protease is present as B-chain (reducing conditions) or γB-fragment (non-reducing) for all variants studied, although it was weak or absent for the CCP1 variant (p.(C338R)), and the γB-fragment was shortened for the CCP2 variant (p.W435R; predicted size: 34 kDa) because of the new cleavage site.

In the next step, mutation p.S654A was inserted in C1r WT and all C1r variants. The mutation Ser654 to Ala destroys the active center of the serine protease domain by itself, independently of any interaction with C1Inh. Therefore, this mutation (p.S654A) prevents auto-activation and subsequent auto-proteolytic cleavage of C1r. In this setting, WT as well as variant p.(D290G) and the CCP2 variant (p.W435R) showed secretion of the non-activated full-length protein in western blots with both, N- and C-terminal specific anti-C1r antibodies. No C1r protein was detected for the other inactivated C1r variants (CUB1, CUB2, CCP1) indicating intracellular retention or degradation (Figures 4E,F).

### All C1r Variants Retain Enzymatic Activity Toward C1s

The only known substrate of C1r is C1s, which is cleaved by activated C1r. Thus, C1s cleavage by C1r WT and variants was determined by two different experimental settings.

**Figure 5:**
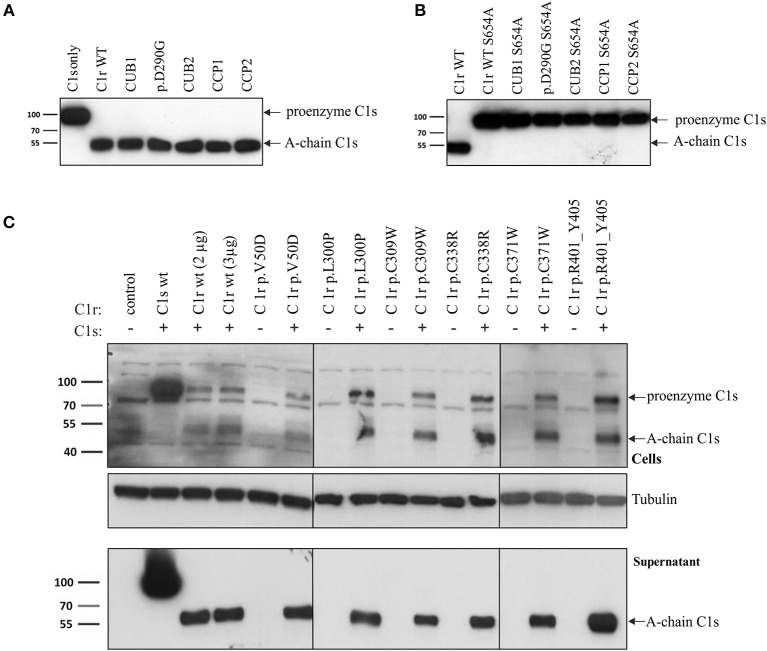
All pEDS C1r variants retain enzymatic function toward C1s. Similarly to C1r, cleavage of proenzyme C1s (90 kDa) produces two disulfide-linked fragments: the C1s A-chain (N-terminal, 55 kDa) and B-chain (C-terminal). Presence of full-length C1s and cleaved C1s (A chain) was studied with an N-terminal anti-C1s antibody under reducing conditions. **(A)** HEK293T cells were transiently transfected with either C1r (WT or mutated) or C1s (WT), and supernatant was collected 48 h post-transfection. C1s supernatant was mixed 1:1 with supernatant from the different C1r overexpressing cells (C1r WT, CUB1, p.(D290G), CUB2, CCP1, CCP2; variants as in Figure 4), incubated for 1 h at 37°C, and used for western blot analysis. C1s cleavage was observed for all cell supernatant mixtures. **(B)** Repeating the analysis after introduction of the inactivating mutation p.S654A into the C1r constructs prevents enzymatic cleavage of C1s in all cases. **(C)** C1r (WT and different variants) was overexpressed either on its own or by co-transfection with C1s WT; C1s in cell lysate (upper panel) or supernatant (lower panel) was visualized by western blot. The results show that all C1r constructs remain activity toward C1s, and that C1r-mediated C1s cleavage already occurs within the cells.

Co-incubation of C1s (WT) and C1r (WT or variant) supernatant showed that all analyzed C1r variants cleaved C1s, whereas uncleaved full-length C1s was present when C1s supernatant was incubated in absence of C1r (Figure 5A). Cleavage is demonstrated by the presence of the C1s A-chain in western blots. Insertion of the inactivating mutation p.S654A into all C1r constructs prevents C1s cleavage (Figure 5B), confirming that C1s activation was due to C1r activity and not to an unspecific protease activity in the supernatants.

In a second approach, C1s was co-expressed with C1r WT or variants in HEK293T cells. Results shown in Figure 5C demonstrate C1s activation in all co-transfection experiments regardless of C1r WT or mutation status. C1s cleavage (albeit partial) was observed in cell lysates, indicating that C1s activation may occur during post-translational processing within the cell.

### Constitutive C1s Activation in Fibroblasts From Individuals With Periodontal EDS

**Figure 6:**
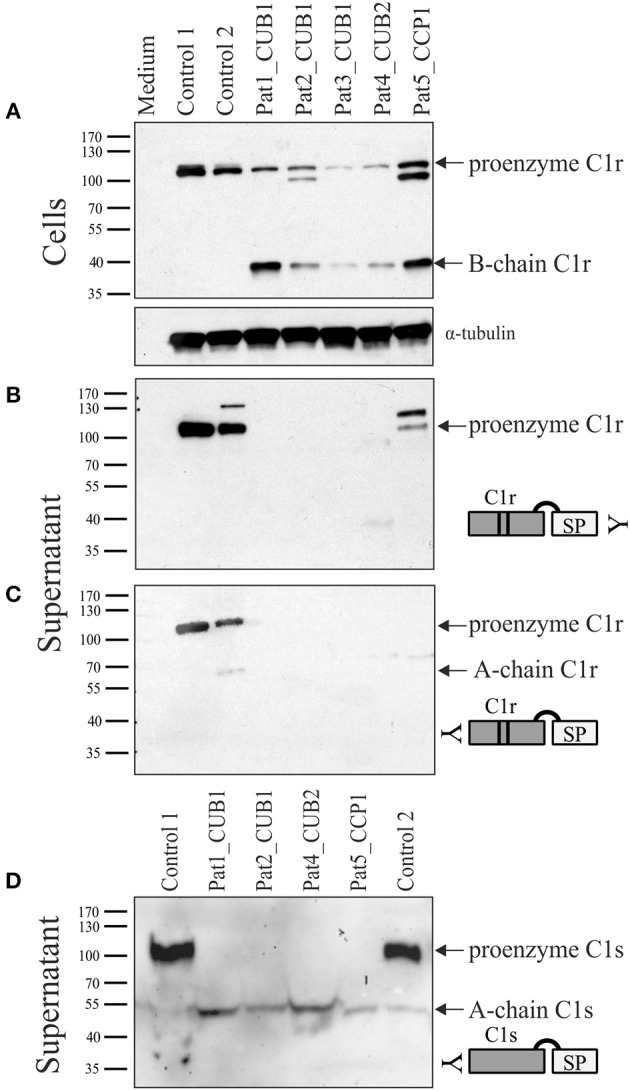
Western blot analyses of patient and control skin fibroblast lysate and supernatant under reducing conditions. Patients 1–3 carry C1r p.V50D (CUB1), patient 4 carries C1r p.R301P (CUB2), and patient 5 carries C1r p.C309F (CCP1). Detection with C-terminal C1r antibody in cell extracts **(A)** and supernatant **(B)**, and with N-terminal C1r **(C)** and C1s **(D)** antibodies in supernatant. Control cells showed uncleaved full-length C1r and C1s protein both within cells **(A)** as well as supernatant **(B–D)**, with little evidence of activation. In patient samples, both full-length and activated C1r (B-chain) were detected in patient cell extracts, but no C1r protein bands were visible in supernatants except at low amounts in patient 5. In contrast to controls, no full-length C1s was present in supernatants of all patient fibroblasts **(D)** indicating complete C1s activation caused by the presence of heterozygous C1r variants. Additional bands in patients 2 and 5 cell extracts (~80 kDa), and control 2 and patient 5 supernatants (~115 kDa) with the C-terminal C1r antibody are non-specific.

Individuals with pEDS caused by heterozygous *C1R* variants co-express WT and variant transcripts in equal amounts, and also co-express C1s and C1Inh. Fibroblasts from five individuals with pEDS (three different patients with the same variant in CUB1, one variant in CUB2 or CCP1) were available for examination of C1r and C1s processing and secretion by western blot with different antibodies. In cell extracts, two different N-terminal C1r antibodies (from abcam: ab71652; ab66751) and two C1s antibodies (abcam: ab155270; ab134928) all showed non-specific additional bands and could not be used for the analyses. The C-terminal anti-C1r antibody (abcam: ab185212) showed C1r-specific bands in cell extracts and supernatants and the anti-C1s antibody (abcam: ab155270) could be used for supernatants (Figure 6). In control cells, C1r is visible as full-length protein (100 kDa) within cell extracts and supernatants with little evidence of activation. In contrast, patient cells showed considerable amounts of activated C1r in cell extracts in conjunction with absent or reduced C1r secretion into the supernatant. Additional bands in patients 2 and 5 cell extracts (approx. 80 kDa), and control 2 and patient 5 supernatants (~115 kDa) with the C-terminal C1r antibody are either non-specific, represent non-glycosylated protein fragments or C1r B-chain in complex with C1Inh. Remarkably, analyses of supernatants with an anti-C1s antibody showed large amounts of proenzyme in control supernatants but complete absence of full-length C1s in patient fibroblast supernatant. In contrast, activated C1s A-chain was present in supernatants of patient fibroblasts but only in very small amounts for control fibroblasts (Figure 6D). This indicates that the presence of variant C1r disturbs secretion of proenzymatic C1r WT in patients with pEDS, compatible with a gain-of-function effect.

### Increased Complement 1 Activation in Fibroblasts From Individuals With Periodontal EDS

**Figure 7:**
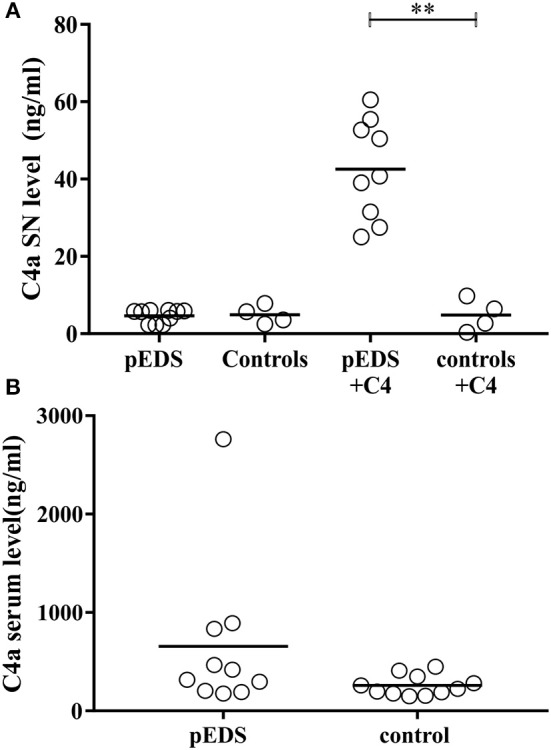
Significantly increased complement 1 activation in fibroblast supernatants from individuals with pEDS. C4a was measured in serum and supernatant as a marker for complement 1 activation. **(A)** After addition of C4 to the supernatant of human-derived skin fibroblast a significant increase of C4 cleavage was detected in pEDS (*n* = 9) but not in controls (*n* = 4) (line indicates median, *p* = 0.003). *P*-values were calculated by Mann-Whitney test (α = 0.05). **(B)** Serum concentrations of C4a were increased in some pEDS patients, but there was no significant overall difference between patients (*n* = 10) and controls (*n* = 11). α = 0.05; ^*^ ≤ 0.05; ^**^ ≤ 0.01; ^***^ ≤ 0.001.

In order to assess a possible activation of the classical complement pathway caused by presence of active C1r variants we investigated the concentrations of activated complement 4 (C4a) in pEDS patient fibroblast cultures and serum samples. It should be noted that cultured skin fibroblasts express C1r, C1s, and C1Inh, but neither C1q nor C4 (22). C1q is required for specific activation of the classical complement pathway (23). C1Inh is the only known regulator of C1r and C1s (24). After addition of C4 to the harvested cell culture supernatant, highly significant C4 activation was detected in fibroblast supernatant of pEDS patients (median 40.79 ng/ml, interquartile range 29.51–54.08). In contrast, values of control fibroblasts (median 4.55 ng/ml, IQR 0.95–8.95) were unchanged compared to baseline in the absence of C4 (Figure 7A, *p* = 0.003). The results imply that activated C1s secreted by patient fibroblasts causes C1q-independent activation of the classical complement pathway. Measurement of systemic C4a concentrations in serum samples from pEDS individuals (median 368.4 ng/ml, IQR 200.5–848.2) and controls (median 223.2 ng/ml, IQR 176.5–349; *p* = 0.07) showed increased variation in patients, but the differences were not significant (Figure 7B). Highly elevated C4a concentrations were observed in one patient with manifest inflammatory bowel disease.

## Discussion

Periodontal EDS is the only known EDS type that is directly linked to the innate immune system (4). Our analyses indicate that pEDS variants cause constitutive intracellular activation of C1s (and C1r) serine proteases resulting in C4 cleavage and local complement cascade activation, as well as other possible consequences. The organization of the complement system as an activation cascade entails an enormous amplification capacity that therefore depends on tight regulation. Permanent immune surveillance and regulation of inflammatory pathways are essential for periodontal homeostasis. The gingival crevicular fluid that contains complement proteins is constantly secreted to keep the periodontal tissue intact (25). Even in the healthy periodontium there are low levels of inflammation mediated by innate and adaptive immune responses (26, 27). Studies demonstrated that C3 activation is a central element in the development of periodontitis in mice (10). Constitutive complement activation is strongly associated with periodontitis because C3-deficient mice (10) as well as C5aR1 antagonist treated mice (28) are protected against periodontitis. Complement inhibitors—such as the C3 complement inhibitor AMY-101 (29)—have been suggested as promising therapeutic options for periodontitis and have already entered clinical trials (30).

**Figure 8:**
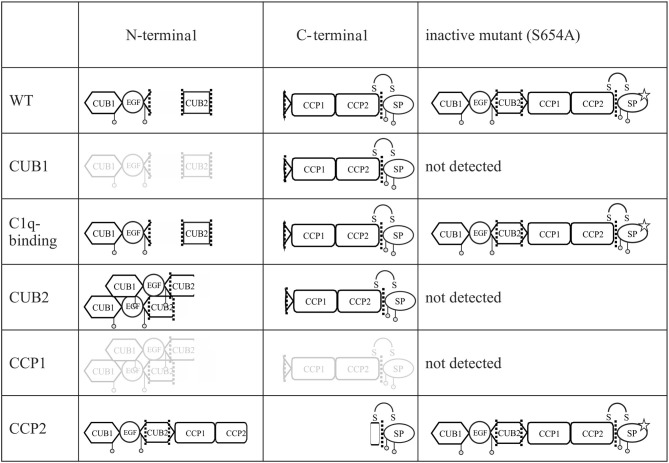
Graphical summary of secreted fragments for all C1r variants. All fragments of enzymatic active and inactive C1r variants in the supernatant of transfected HEK293T cells and detected on western blot by N- and C-terminal anti-C1r antibody are presented. Fragments presented in gray color indicate faint bands on western blot. Mutation p.S654A is marked by a star. Putative aggregate formation for CUB2 mutants is indicated.

Since pEDS is a rare genetic disease, access to patient samples was limited. In addition, patient-derived fibroblasts express both C1r WT and C1r variant without possibility to distinguish between them. We therefore decided to establish an *in-vitro* model for a more detailed examination of intracellular processing and secretion of WT C1r and the different individual variants. Overexpression of C1r in HEK293T cells differs from the physiological context as HEK293T cells do not assemble C1 complement proteins and do not produce C1Inh required for the inhibition of C1r auto-cleavage. Transfected C1r WT in HEK293T cells was thus expected to show intracellular activation (Figure 2), in contrast to the proenzyme C1r WT secreted by control human-derived skin fibroblasts (Figure 6). A graphical summary is provided in Figure 8. The experiments allowed two main conclusions:

1. Different pEDS variants have very different but domain-specific effects on processing and secretion (Figure 2). There was no evidence to suggest that specific intracellular processing abnormalities or interactions of the N-terminal domains (A-chain) has specific pathogenic effects in pEDS.
2. All *C1R* variants result in the production of enzymatically active serine protease (B-chain) which is secreted (Figure 4). Auto-activated C1r serine protease is able to activate C1s (Figure 5).

The majority of pEDS variants showed intracellular retention (Figure 2), explaining the apparent extension of the endoplasmic reticulum after overexpression of pEDS variants in HEK293T cells (4). The C-terminal catalytic γB-chain is effectively secreted and will additionally cleave proenzymatic C1r WT that may exert pathogenic extracellular effects. Extracellular A-chain of variants was either not visualized or differed in fragment size from C1r WT on western blot, except p.(D290G) (Figure 2). A-chain is expected to be in the supernatant because γB-chain was visualized and auto-proteolytic cleavage—separating αβ- from γB-chain (positions Arg228 and Arg296)—can only take place extracellularly in the presence of Ca^2+^. Thus, αβ-fragments are present in the supernatant but aggregate thereby hiding the C1r-antibody epitope which results in low signal intensity on western blot. Interestingly, prevention of C1r auto-activation by introduction of the active center mutation p.S654A did not interfere with secretion of uncleaved C1r WT, the C1q interaction variant p.(D290G), or the variants that introduce a new auto-activation site in the CCP2 domain. In contrast, all other pEDS variants co-expressed with p.S654A showed intracellular retention of the uncleaved protein, indicating substantial structural alterations that interfere with normal post-translational processing. It cannot be excluded that retained intracellular C1r fragments have adverse effects e.g., on the processing of ECM components, but this needs further investigation.

The X-ray crystal structure of C1r CUB2 module has been solved recently, allowing identification of acidic residue D290 as a calcium ligand (31). In addition, our previous mutagenesis studies had shown that this amino acid is involved in the interaction of the C1s-C1r-C1r-C1s tetramer with C1q (21). We therefore assumed that the substitution by a non-polar glycine (p.(D290G)) would also inhibit binding of the mutated tetramer to C1q, which was confirmed by SPR analyses (Figure 3). Whether the C1q binding variant p.(D290G) also triggers abnormal intra- or extracellular C1r and C1s activation remains to be confirmed through fibroblast studies from pEDS patients with this variant, which were not available up to now. One possible pathomechanism of p.(D290G) may be abnormal release of enzymatically active C1r/C1s tetramer from the complement 1 complex, but increased intracellular C1r auto-activation e.g., due to reduced C1Inh binding cannot be excluded.

C1r variant p.W435R (CCP2 domain) introduces a new cleavage site at amino acid position 401. This residue likely becomes exposed because of structural changes induced by the p.W435R variant, potentially causing partial unfolding of the CCP2 module. Destabilization of the dimeric catalytic domain of C1r would result in the absence of cleavage at the usual A-chain auto-proteolysis sites. Interestingly, the second CCP2 variant [p.(Arg401_Tyr405delinsHisValIle)], which yields a similar C1r expression profile (Figure 2), results in deletion of Arg401. However, it is likely that removal of a 5-residue segment in CCP2 also results in unfolding of this domain, which might expose other potential cleavage sites at Arg or Lys residues located in the vicinity, such as Lys399. Getting a definite answer would require production and purification of the corresponding mutated C1r [p.(Arg401_Tyr405delinsHisValIle)], a time-consuming procedure due to the low amounts of mutated protein secreted.

Some mutated N-terminal C1r fragments (CUB2 variants) show a tendency to associate as dimers or to aggregate extracellularly (Figure 2) due to increased Ca^2+^ concentrations. CUB domains contain conserved β-sheets which are involved in formation of aggregates (32, 33). It is plausible that extensive structural changes due to C1r variants alter the arrangement of β-sheets and facilitate aggregation, although the exact nature of this cannot be specified. As expected, western blot analysis of the putative control variant p.(R301C) showed the same pattern as WT C1r, in contrast to the pEDS variant p.R301P (Figure 2). The substitution of an arginine by a proline residue likely affects the local structure of the CUB2 domain. Covalent aggregation of mutated CUB domains combined with reduced secretion into the cell culture medium has also been noticed for the metalloprotease ADAMTS-13 (34). Moreover, the four C1r CUB2 variants with this secretion pattern are located in close proximity of the auto-proteolytic cleavage site at Arg296. While this cleavage occurs following the first auto-proteolytic cleavage at Arg228 upon incubation of purified serum-derived C1r at 37°C (6), it might be favored by the variants and the resulting αβ-fragment would yield a band at about 40 kDa. The higher molecular weight band could correspond to dimers of this fragment. Indeed the isolated CUB1-EGF-CUB2 fragment has been shown to form dimers (31) that might be resistant to reducing conditions.

Because of the absence of C1Inh in HEK293T cells, it remained unclear whether the specific effects observed for the different variants also occur under physiological conditions. C1Inh has been shown to interact with the catalytic site of C1r in the extracellular space (24). However, there have been no studies on the possible intracellular interaction of C1Inh with C1r or C1s. The three proteins are usually co-expressed in the same cell types. Considering the high auto-catalytic activity of C1r it is likely that co-expression with C1Inh is required for production and secretion of full-length C1r (and C1s together with C1r). Interestingly, dysfunctional C1Inh was reported in a 24 year old woman with an apparently aggressive form of periodontitis and severe angioedema restricted to the gingiva, although the molecular basis remained uncertain (35). A systematic coexpression analysis of the interaction of C1r (and its variants) with C1inh was beyond the scope of our study. Examination of available fibroblast samples from pEDS patients showed that pathogenic C1r variants trigger abnormal intracellular activation of C1r, whereas activation is prevented presumably by presence of the co-expressed C1Inh in control cells (Figures 6A,B). This effect should be confirmed by more detailed kinetic analyses for all C1r variants in future studies. Normal expression of C1Inh in patient fibroblasts was confirmed by qPCR, and genomic sequence analyses did not reveal any putative functional variants in the *SERPING1* gene which codes for C1Inh (data not shown). Absent or hardly detectable fragments of C1r in the supernatant of patient skin fibroblasts (Figure 6C) most likely indicate intracellular retention or degradation of misfolded activated protein. In pEDS, C1r activation also triggers complete C1s activation with a lack of C1s proenzyme in the supernatant, representing a gain-of-function effect (Figure 6D). C1r-C1s interaction via N-terminal domains is not necessary for extracellular C1s activation, as observed previously for the recombinant catalytic γB-fragment of C1r (36, 37). When complement factor C4 was added to the supernatant of pEDS and control fibroblast, C4 activation in the absence of external triggers was observed only for patient cells and not for control cells (Figure 7A).

The release of activated C1s by C1r/C1s/C1Inh producing cells e.g., in the periodontal region may result in local dysregulation of the complement cascade, as well as further uncontrolled cleavage of other target(s) that still need to be investigated. Numerous components or modulators of the extracellular matrix (ECM) contain CUB domains (33) and are possible interaction partners or substrates of mutated C1r or constitutively activated C1s. There was no significant difference in systemic (serum) C4a concentrations between patients and controls. One patient showed very high levels of C4a, but this is well-explained by active inflammatory bowel disease in this individual. These data are in line with clinical presentation of pEDS patients who do not show symptoms of generalized systemic complement activation. This does not exclude the possibility that some clinical features of pEDS are linked to increased responsiveness to triggers of complement activation in the periodontal tissue and possibly other organs under certain conditions.

In conclusion, we report that all known C1r variants causing pEDS have two complement-associated features in common: (a) they result in uncontrolled cleavage of C1s and thus a gain-of-function for uncontrolled protease activity against C4 and potentially unspecified other targets; (b) they are incapable of forming the C1 complex because of missing C1q binding site, extensive structural alterations, or missing interaction domains. pEDS thus seems to be a disorder of constitutive local complement cascade activation in the periodontal region and possible other effects on ECM proteins in organs with complement 1 expression.

## Data Availability Statement

All datasets generated for this study are included in the article/Supplementary Material.

## Ethics Statement

The studies involving human participants were reviewed and approved by Ethics Committee of the Medical University Innsbruck (UN4501 and 1074/2017). The patients/participants provided their written informed consent to participate in this study.

## Author Contributions

RG performed the wet lab work, data analysis, and wrote the manuscript draft. IK-S contributed to the study design, examined patients, and took samples. AA supervised the wet lab work. RR performed western blot analyses of Figure 2. EB and DM contributed patient material and revised the manuscript. CG and NT contributed to study design. FD and IB performed the experiments with purified C1r variants. CG, IB, NT, and VR contributed to data analysis. CG, VR, and NT corrected the draft of the manuscript. HS contributed to data analysis of serum analyses. JZ contributed to study design, supervised and coordinated the project, and corrected the manuscript. All authors contributed to manuscript revision, read and approved the submitted version.

### Conflict of Interest

The authors declare that the research was conducted in the absence of any commercial or financial relationships that could be construed as a potential conflict of interest.

## Supplementary Material

Figure S1 Cell lysates and supernatants of HEK293T cells with C-terminal antibody. HEK293T cells were transiently transfected with C1r WT or variants (“control” = empty vector). Cell lysates (upper panel) and supernatants (lower panel) were used for western blot with an C-terminal anti-C1r antibody 48 h after transfection under reducing conditions. This antibody may specifically visualize the full-length C1r (100 kDa) and the B-chain (38 kDa) after auto-activation of C1r. Little full-length C1r and presence of B-chain was detected in all cell lysates. C1r WT and all C1r variants resulted in extracellular presence of B-chain. For CCP1 mutants hardly visible bands representing the B-chain were detected.
