# Corrigendum: *C1R* Mutations Trigger Constitutive Complement 1 Activation in Periodontal Ehlers-Danlos Syndrome

**DOI:** 10.3389/fimmu.2019.02837

**Published:** 2019-12-04

**Authors:** Rebekka Gröbner, Ines Kapferer-Seebacher, Albert Amberger, Rita Redolfi, Fabien Dalonneau, Erik Björck, Di Milnes, Isabelle Bally, Veronique Rossi, Nicole Thielens, Heribert Stoiber, Christine Gaboriaud, Johannes Zschocke

**Affiliations:** ^1^Institute for Human Genetics, Medical University Innsbruck, Innsbruck, Austria; ^2^Department for Operative and Restorative Dentistry, Medical University Innsbruck, Innsbruck, Austria; ^3^University of Grenoble Alpes, CEA, CNRS, IBS, Grenoble, France; ^4^Department of Molecular Medicine and Surgery, Karolinska Institute, Stockholm, Sweden; ^5^Department of Clinical Genetics, Karolinska University Hospital, Stockholm, Sweden; ^6^Genetic Health Queensland, Royal Brisbane and Women's Hospital, Herston, QLD, Australia; ^7^Institute of Virology, Medical University Innsbruck, Innsbruck, Austria

**Keywords:** complement system, connective tissue, periodontitis, C1r/s, Ehlers-Danlos syndrome

In the original article, there was an error. “Mag. Brigitte Müllauer” erroneously omitted from the **Acknowledgments** section.

A correction has been made to **Acknowledgments**:

“Special thanks to Mag. Brigitte Müllauer for her valuable support and practical contributions. We thank Jean-Pierre Andrieu and Guillaume Fouet for performing N-terminal sequence determination and SPR analyses, respectively. We are grateful to Robert James McKinlay Gardner (Genetic Health Queensland, Royal Brisbane and Women's Hospital, Herston, Australia) and Hannah Burns (School of Medicine, University of Queensland, Brisbane, Australia and Department of Otolaryngology Head and Neck Surgery, Lady Cilento Childrens Hospital, Brisbane, QLD, Australia) for kindly providing precious patient material. We thank the reviewers for helpful comments and suggestions during the review process. We thank all the patients and their families who contributed to the study.”

The authors apologize for this error and state that this does not change the scientific conclusions of the article in any way. The original article has been updated.

