# Supplementary figures and images for: C1R Mutations Trigger Constitutive Complement 1 Activation in Periodontal Ehlers-Danlos Syndrome
Source: Front Immunol. 2019 Nov 5;10:2537. doi: 10.3389/fimmu.2019.02537 (PMC6848165; doi:10.3389/fimmu.2019.02537)

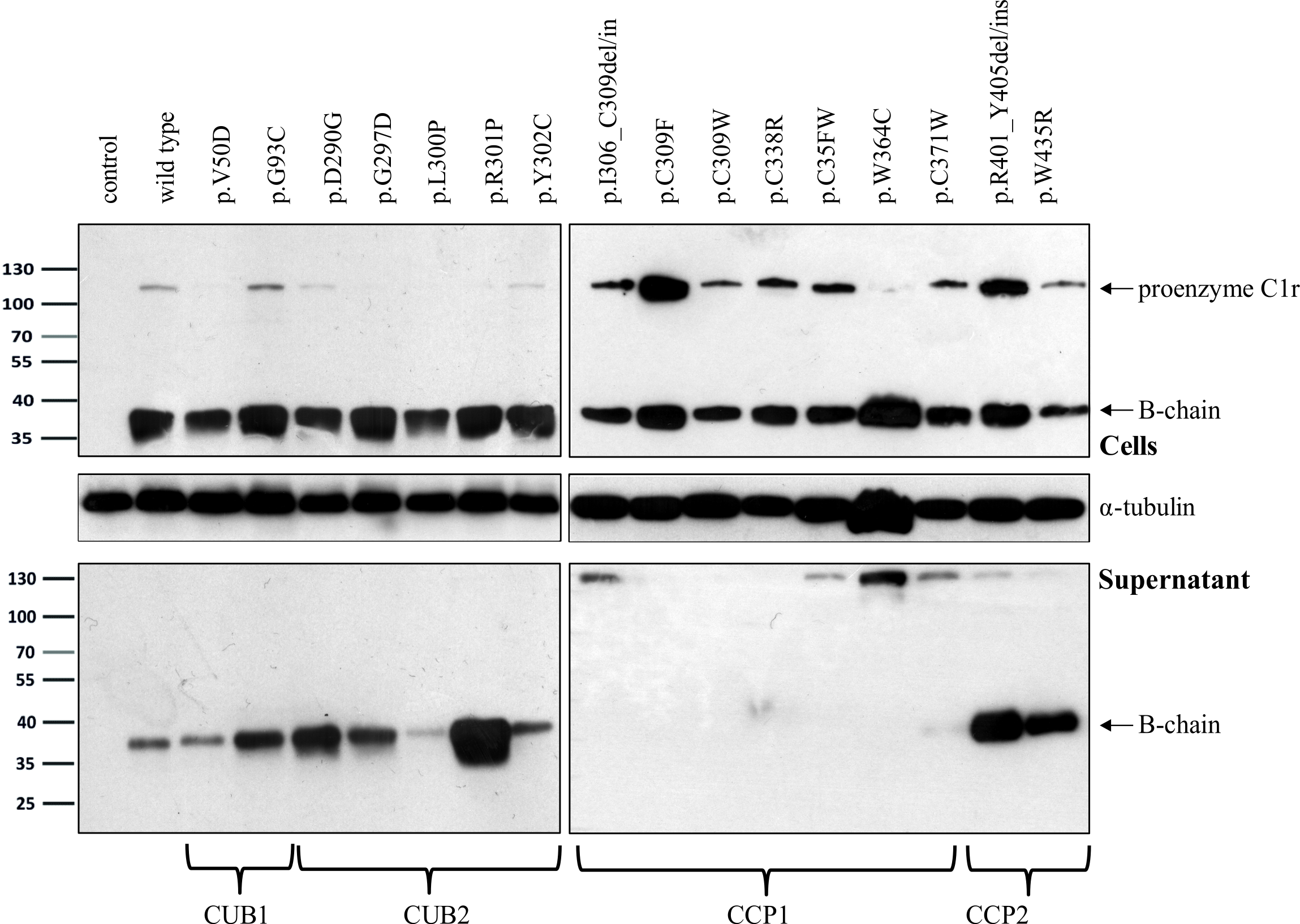

Supplement: Figure S1 — Cell lysates and supernatants of HEK293T cells with C-terminal antibody. HEK293T cells were transiently transfected with C1r WT or variants (“control” = empty vector). Cell lysates (upper panel) and supernatants (lower panel) were used for western blot with an C-terminal anti-C1r antibody 48 h after transfection under reducing conditions. This antibody may specifically visualize the full-length C1r (100 kDa) and the B-chain (38 kDa) after auto-activation of C1r. Little full-length C1r and presence of B-chain was detected in all cell lysates. C1r WT and all C1r variants resulted in extracellular presence of B-chain. For CCP1 mutants hardly visible bands representing the B-chain were detected. [file Image_1.tif]
